# CDDO-Me Inhibits Microglial Activation and Monocyte Infiltration by Abrogating NFκB- and p38 MAPK-Mediated Signaling Pathways Following Status Epilepticus

**DOI:** 10.3390/cells9051123

**Published:** 2020-05-01

**Authors:** Ji-Eun Kim, Hana Park, Ji-Eun Lee, Tae-Cheon Kang

**Affiliations:** Department of Anatomy and Neurobiology, Institute of Epilepsy Research, College of Medicine, Hallym University, Chuncheon, Kangwon-Do 24252, Korea; jieunkim@hallym.ac.kr (J.-E.K.); M19050@hallym.ac.kr (H.P.); 20183533@hallym.ac.kr (J.-E.L.)

**Keywords:** CD68, epilepsy, IB4, Iba-1, Nrf2, SN50, seizure

## Abstract

Following status epilepticus (SE, a prolonged seizure activity), microglial activation, and monocyte infiltration result in the inflammatory responses in the brain that is involved in the epileptogenesis. Therefore, the regulation of microglia/monocyte-mediated neuroinflammation is one of the therapeutic strategies for avoidance of secondary brain injury induced by SE. 2-cyano-3,12-dioxooleana-1,9-dien-28-oic acid methyl ester (CDDO-Me; RTA 402) is an activator of nuclear factor-erythroid 2-related factor 2 (Nrf2), which regulates intracellular redox homeostasis. In addition, CDDO-Me has anti-inflammatory properties that suppress microglial proliferation and its activation, although the underlying mechanisms have not been clarified. In the present study, CDDO-Me ameliorated monocyte infiltration without vasogenic edema formation in the frontoparietal cortex (FPC) following SE, accompanied by abrogating monocyte chemotactic protein-1 (MCP-1)/tumor necrosis factor-α (TNF-α) expressions and p38 mitogen-activated protein kinase (p38 MAPK) phosphorylation. Furthermore, CDDO-Me inhibited nuclear factor-κB (NFκB)-S276 phosphorylation and microglial transformation, independent of Nrf2 expression. Similar to CDDO-Me, SN50 (an NFκB inhibitor) mitigated monocyte infiltration by reducing MCP-1 and p38 MAPK phosphorylation in the FPC following SE. Therefore, these findings suggest, for the first time, that CDDO-Me may attenuate microglia/monocyte-mediated neuroinflammation via modulating NFκB- and p38 MAPK-MCP-1 signaling pathways following SE.

## 1. Introduction

Microglia are unique non-neuroepithelial and myeloid cells found in the brain parenchyma as the brain is separated from the systemic immune system by a brain-blood barrier (BBB) [1]. Under physiological conditions, microglia have small cell bodies with slender ramified processes (resting states). In response to various stresses, microglia show hypertrophic and/or elongated morphologies with hyper-ramified processes (intermediate states) and finally amoeboid shape indicating phagocytic and cytotoxic activity (activated states) [2]. Microglial activation is mediated by various receptors and ion channels (such as purinergic receptors, C-X3-C motif chemokine receptor, transient receptor potential channel (TRPC) and K^+^ channels), which activate diverse signaling molecules including the nuclear factor of activated T-cells, nuclear factor-κB (NFκB), mitogen-activated protein kinases (MAPKs) and phosphatidylinositol-3-kinase (PI3K)/AKT [3]. This microglial activation seems to play an important role in epileptogenic processes. Activated microglia secrete various cytokines such as interleukin-1β (IL-1β) and tumor necrosis factor-α (TNF-α), which evoke neuronal hyper-excitability and the neuronal damage [4,5,6]. Activated microglia also regulate synaptic activity by engulfing pre- or post-synapses. This aberrant microglia-dependent synaptic pruning changes the excitation/inhibition balance of neurons toward excitation, which leads to the development and aggravation of epilepsy [7]. Furthermore, activated microglia leads to the recruitment of neutrophils and monocytes from blood into the brain parenchyma by various chemokines such as monocyte chemotactic protein-1 (MCP-1) that is a primary chemokine to recruit monocyte/macrophage-mediated by C-C motif chemokine receptor 2 (CCR2) [8,9,10]. Together with activated microglia, infiltrating leukocytes accelerate local inflammatory processes through the generation of toxic free radicals, the release of proteolytic enzymes, and synthesis of pro-inflammatory cytokines [11,12,13]. Therefore, the regulations of microglial activation and blood-derived leukocyte infiltration are one of the primary therapeutic strategies for the inhibition of undesirable consequences (including epileptogenesis) from brain insults. 

Status epilepticus (SE, a prolonged seizure activity) is a neurologic emergency and one of the risk factors of developing acquired epilepsy. SE leads to microglial activation that initiates cytokine-mediated inflammatory responses, which evoke the alterations in neuronal excitability, neuronal loss, and aberrant neurogenesis [14,15]. Indeed, anti-epileptic drugs (AEDs) attenuate neuroinflammation induced by seizure activities and other harmful stresses [16,17,18,19,20,21,22,23] through the inhibition of cyclooxygenase-2 expression by increasing MAPK phosphatase-1 (MKP-1) activity [20], inactivation of microglial Na_v_ 1.6 channel [17], suppression of TNF-α and IL-1β inductions [16,21,22] and blockade of NFκB signaling pathway [18,23]. In addition to microglial activation, SE results in blood-derived leukocyte infiltration in brain parenchyma with/without BBB disruption [8,24]. For example, SE leads to leukocyte infiltration in the piriform and entorhinal cortices with vasogenic edema via the impaired BBB integrity. In contrast, SE induces it in the frontoparietal cortex (FPC) without BBB breakdown [8,25]. In both regions, neutrophils transiently appear in brain parenchyma during the acute phase of SE (4–36 h after SE) disappearing thereafter. Later, monocytes are found in brain parenchyma and persist during the epileptogenic (latent) period [8,14]. Infiltrating monocytes lead to the sustained neuronal injury by releasing cytotoxic mediators, which subsequently induces the pathological sequelae of specific epileptogenic foci [14,26]. Therefore, microglial activation and monocyte infiltration may contribute to epileptogenesis induced by SE. 

2-cyano-3,12-dioxooleana-1,9-dien-28-oic acid methyl ester (CDDO-Me; bardoxolone-methyl; RTA 402) is a triterpenoid analog of oleanolic acid that has pleiotropic effects. At low (nanomolar) doses, CDDO-Me shows cell-protective effects against oxidative stress by activating nuclear factor-erythroid 2-related factor 2 (Nrf2) that modulates intracellular redox homeostasis by regulating the levels of reactive oxygen species (ROS) [27,28]. In addition, Nrf2 inhibits inflammation by reducing the expression of proinflammatory cytokines, including TNF-α and IL-1β [29]. In vivo antioxidant and anti-inflammatory doses of CDDO-Me are ~0.05 nmol/kg/day intracerebroventricular (i.c.v.) infusion over 7 days [30,31] and 0.4–4 μmol/kg once-intravenous injection (i.v.) [28,32]. At doses higher than 100 nM, CDDO-Me modulates cell differentiation, and at micromolar doses, it exerts cytotoxic, anti-proliferative, pro-apoptotic, and anti-cancer effects [27,33] as the higher concentrations of CDDO-Me affect PI3K/Akt/mammalian target of rapamycin (mTOR), c-Jun NH_2_-terminal kinase and Janus-activated kinases signaling pathways [29,34,35]. The anti-proliferative and pro-apoptotic activities of CDDO-Me are generally observed at a concentration from 0.1 to 10 μM and above 0.5 μM, respectively [29,34,35]. In vivo anti-cancer doses of CDDO-Me are at least 15 μmol/kg/day per oral (p.o.) over 7 or 20 weeks [29,34,35]. In addition, CDDO-Me directly inhibits NFκB signaling at concentrations with 0.25–1 μM in U-937 myeloid leukemia cells [36] and 1.25–10 μM in prostate cancer cells [37]. Unlike cancer cells, CDDO-Me (10–100 nM) inhibits expressions of pro-inflammatory gene, toll-like receptor, and nitric oxide synthase in the leukocytes, macrophage, and microglia without cytotoxicity [27,38], which are regulated by NFκB signaling pathway [31,39,40]. In addition, CDDO-Me (<50 nM)-mediated Nrf2 activation reduces MCP-1 production in human blood immune cells induced by lipopolysaccharide (LPS) [41]. CDDO-Me also suppresses microglial proliferation and its activation at a concentration of 0.4 nM in vitro and ~0.5 nmol/kg/day (i.c.v.) over 7 days in vivo [31,40]. Thus, the anti-cancer concentrations of CDDO-Me in vitro and in vivo are ~250–25,000 and 30,000 times higher than anti-inflammatory doses, respectively. Thus, it is noteworthy to explore the effects of CDDO-Me on microglial activation and monocyte infiltration following SE, although once-daily administration of 20 mg of CDDO-Me increases the risk of the cardiovascular dysfunctions in patients with prior history of heart failure without evidence of direct cardiotoxicity [42,43,44].

Here, we demonstrate that CDDO-Me effectively attenuated SE-induced microglial activation and monocyte infiltration in the FPC by inhibiting NFκB- and p38 mitogen-activated protein kinase (p38 MAPK)-mediated inductions of MCP-1 and TNF-α, independent of Nrf2 activity. Therefore, our findings propose an underlying anti-inflammatory mechanism of CDDO-Me by regulating microglial functions, and its availability for neuroinflammation.

## 2. Materials and Method

### 2.1. Experimental Animals and Chemicals

Adult male Sprague–Dawley (SD) rats (7 weeks old) were used in the present study. Rats were in-housed under controlled conditions (22 ± 2 °C, humidity 55 ± 5%, a light-dark cycle on a 12-h on-off cycle) and freely accessed to water and food throughout the experiments (See Supplementary Materials). All experimental protocols were approved by the Institutional Animal Care and Use Committee of Hallym University (Hallym 2018-2, April 2018). All reagents were obtained from Sigma-Aldrich (St. Louis, MO, USA), except as noted.

### 2.2. Surgery and Drug Infusion

Based on our previous studies [30,31], we applied CDDO-Me at a concentration with 10 μM via i.v.c. infusion with 1007D osmotic pump (reservoir volume 100 µL) that supplies 0.5 µL/h of material for 1 week to exclude the possibilities of the poor BBB permeability, inconstant level, and cardiovascular side-effect of CDDO-Me. Thus, each animal was given CDDO-Me at a concentration of ~0.5 nmol/kg/day over 7 days that is 1/30,000 of the minimum anti-cancer (cytotoxic) dose (15 μmol/kg/day, p.o.) in vivo [29,34,35]. A brain infusion kit 1 (Alzet, Cupertino, CA, USA) was implanted into the right lateral ventricle (1 mm posterior; 1.5 mm lateral; 3.5 mm depth) under Isoflurane anesthesia (3% induction, 1.5–2% for surgery and 1.5% maintenance in a 65:35 mixture of N_2_O:O_2_), as previously described [25,30,31,45]. The brain infusion kit was connected to an osmotic pump (1007D, Alzet, Cupertino, CA, USA). Each osmotic pump contained (1) vehicle, (2) CDDO-Me (10 μM), or (3) SN50 (a NFκB inhibitor; 20 μM), which could not result in behavioral and neurological defects in normal animals, and the alterations in seizure susceptibility and its severity in response to pilocarpine [25,30,31,45].

### 2.3. SE Induction 

Three days after surgery, rats were treated with atropine methylbromide (5 mg/kg, i.p.). Twenty min after atropine treatment, rats were given pilocarpine (380 mg/kg, i.p.). Two h after onset of SE, diazepam (Valium; Hoffman la Roche, Neuilly sur-Seine, France; 10 mg/kg, i.p.) was administered. Age-matched controls received the same volume of saline instead of pilocarpine. 

### 2.4. Tissue Processing and Immunohistochemistry

Three days after SE, we transcardially perfused animals with 0.9% saline followed by 4% paraformaldehyde in 0.1M phosphate buffer (PB, pH 7.4) under urethane anesthesia (1.5 g/kg, i.p.), since this time point is the best to observe microglial activation and monocyte infiltration [8,25]. The brains were removed and post-fixed in the same fixative overnight. Next, the brains were stored in 30% sucrose/0.1M PBS for cryoprotection. Coronal sections were made at a 30-μm thickness with a cryo-microtome. After 3 times-wishing with PBS (0.1M, pH 7.3), tissues were incubated in 3% H_2_O_2_ and 10% methanol in PBS (0.1 M) for 20 min at room temperature. Later, sections were incubated in 0.1% bovine serum albumin and subsequently primary antibody (Table 1). Tissue sections were developed in 3,3′-diaminobenzidine in 0.1M Tris buffer and mounted on gelatin-coated slides. Some sections were reacted with a cocktail solution containing the primary antibodies or isolectin B4 (IB4, Table 1) in PBS containing 0.3% Triton X-100 and visualized with appropriate Cy2- and Cy3-conjugated secondary antibodies. A negative control test was carried out with preimmune serum instead of the primary antibody, which showed no immunoreactivity in any structures. 

### 2.5. Cell Count and Measurements of Iba-1 Positive Area and Fluorescent Intensity

For cell counts, sections (10 sections per each animal) were captured and areas of interest (1 × 10^4^ μm^2^) were selected from the FPC using an AxioImage M2 microscope [8,25]. Thereafter, cell counts and ionizing calcium-binding adaptor molecule 1 (Iba-1) positive area were performed using AxioVision Rel. 4.8 Software. For measurement of fluorescent intensity, 30 areas/rat (300 μm^2^/area) were randomly selected within the FPC (15 sections from each animal, *n* = 7 in each group), and mean fluorescence intensities (a 256 grayscale) were measured using AxioVision Rel. 4.8 software (Carl Zeiss Korea, Seoul, South Korea). Fluorescent intensity was normalized by setting the mean background obtained from five image inputs. 

### 2.6. Western Blot

For Western blot, animals were decapitated under urethane anesthesia (1.5 g/kg, i.p.). The FPC was rapidly dissected out and homogenized in lysis buffer. After the measurement of the protein concentration using a Micro BCA Protein Assay Kit (Pierce Chemical, Dallas, TX, USA), standard Western blot was performed (*n* = 7 in each group) using each primary antibody (Table 1). The band was detected and quantified using ImageQuant LAS4000 system (GE Healthcare Korea, Seoul, South Korea). The values of each sample were normalized with the amount of β-actin. The ratio of phospho-protein to total protein was described as the protein phosphorylation level.

### 2.7. Data Analysis

Comparisons between groups were performed using Student *t*-test and one-way ANOVA followed by Bonferroni’s post hoc comparisons. A *p*-value of less than 0.05 was considered to be significant. SPSS 18.0 software was used for all analyses.

## 3. Results

### 3.1. CDDO-Me Influences Monocyte Infiltration and Microglial Morphogenesis Induced by SE

**Figure 1:**
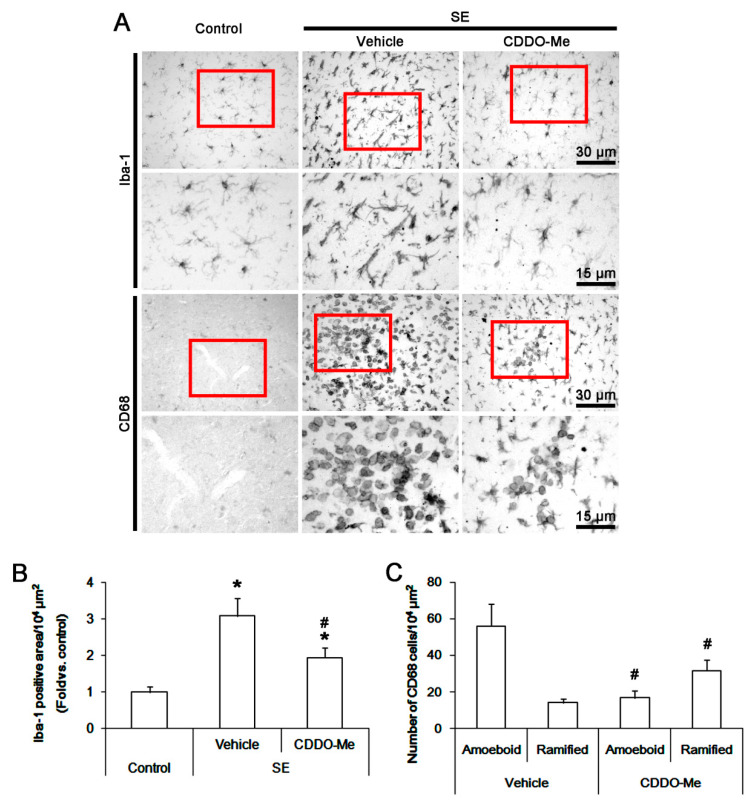
The effect of 2-cyano-3,12-dioxooleana-1,9-dien-28-oic acid methyl ester (CDDO-Me)CDDO-Me on monocyte infiltration and microglia activation in FPC following SE. Iba-1 microglia show hypertrophic/elongated morphologies with hyper-ramified processes that are covered by a lot of thorny spine following SE. Amoeboid or round-shaped CD68 cells are detected following SE. CD68 cells also exhibit hyper-ramified shapes. CDDO-Me attenuates Iba-1 microglia transformation. In addition, CDDO-Me reduces the number of CD68 amoeboid cells but increases that of CD68 hyper-ramified cells. (**A**) Representative images for Iba-1 and CD68 positive cells. (**B**,**C**) Quantification of the effect of CDDO-Me on Iba-1 positive area (**B**) and the number of CD68 amoeboid and ramified cells (**C**) and following SE. Error bars indicate SEM (*^,#^ p < 0.05 vs. control and vehicle, respectively; *n* = 7, respectively).

In control animals, Iba-1 microglia had small cell bodies with thin ramified processes in the FPC. Following SE, Iba-1 microglia were transformed to hypertrophic and/or elongated cell bodies (Figure 1A). Thus, the Iba-1 positive area was increased to 3.1 ± 0.2-fold of control level (*p* < 0.05 vs. control, one-way ANOVA, *n* = 7, respectively; Figure 1B). CDDO-Me reduced the Iba-1 positive area to 1.9 ± 0.3-fold of control level in the FPC following SE (*p* < 0.05 vs. vehicle, one-way ANOVA, *n* = 7, respectively; Figure 1B). 

Few CD68 cells were observed in the FPC of control animals (Figure 1A). Amoeboid/round shaped-CD68 cells were detected in the FPC following SE. The number of amoeboid/round shaped-CD68 cells was 56.1 ± 12.3/10^4^ μm^2^ (Figure 1A,C). CD68 cells were localized in perivascular regions within the FPC. Some CD68 cells showed hyper-ramified shapes. The number of these cells was 14.1 ± 2.3/10^4^ μm^2^. CDDO-Me resulted in ~30% reductions in the number of amoeboid/round shaped-CD68 cells (17 ± 3.9/10^4^ μm^2^) with ~2-fold increase in that of hyper-ramified-CD68 cells (31.7 ± 5.9/10^4^ μm^2^) following SE (*p* < 0.05 vs. vehicle, Student *t*-test, *n* = 7, respectively; Figure 1C). As CD68 is a commonly used marker for peripheral monocytes as well as activated microglia [8,25,46], these findings indicate that CDDO-Me may abrogate the SE-induced microglial activation and monocyte infiltration into the FPC. 

### 3.2. CDDO-Me Mitigated Monocyte Infiltration by Inhibiting Microglial MCP-1 Production Following SE

**Figure 2:**
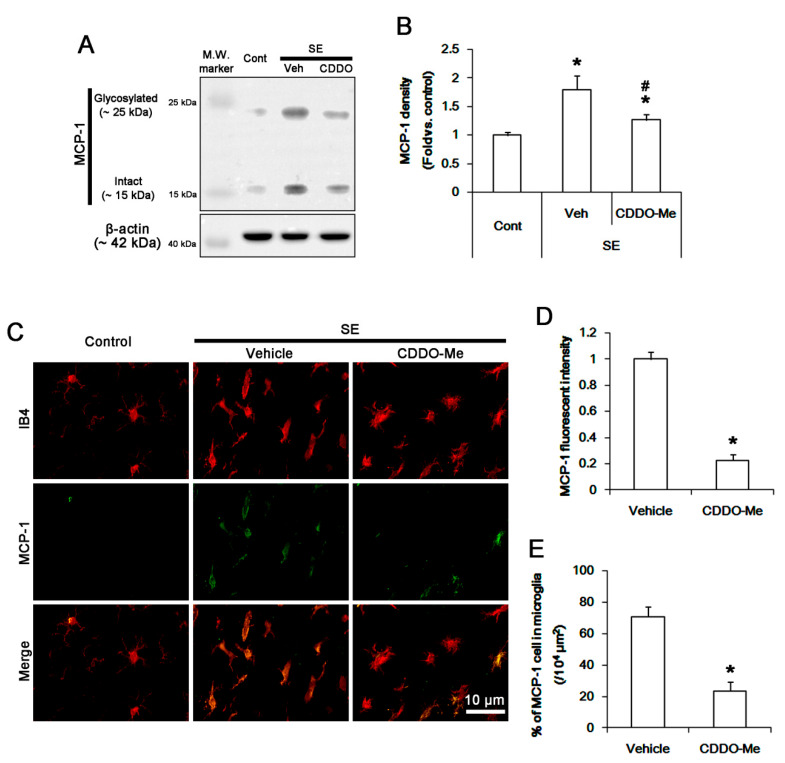
The effect of CDDO-Me on microglial MCP-1 induction after SE. SE increases MCP-1 expression in the FPC, which is attenuated by CDDO-Me. MCP-1 expression is up-regulated in resident IB4 microglia following SE, while it is rarely detected under physiological condition. CDDO-Me diminishes MCP-1 expression in microglia. (**A**) Representative Western blot image for MCP-1. (**B**) Quantification of the effect of CDDO-Me on MCP-1 expression based on Western blot data. Error bars indicate SEM (*^,#^ *p* < 0.05 vs. control and vehicle, respectively; *n* = 7, respectively). (**C**) Representative images for MCP-1 expression in IB4 microglia. (**D**,**E**) Quantification of the effect of CDDO-Me on MCP-1 expression (**D**) and the fraction of MCP-1 positive cells in total microglia (**E**) following SE. Error bars indicate SEM (* p < 0.05 vs. vehicle; *n* = 7, respectively).

Next, we explored whether CDDO-Me affects microglial MCP1 expression following SE. Western blot data revealed that SE increased MCP-1 protein level to 1.8 ± 0.2-fold of control level in the FPC (*p* < 0.05 vs. control, one-way ANOVA, *n* = 7, respectively; Figure 2A,B). CDDO-Me attenuated the SE-induced MCP-1 up-regulation to 1.3 ± 0.1-fold of control level (*p* < 0.05 vs. vehicle, one-way ANOVA, *n* = 7, respectively; Figure 2A,B). Under physiological conditions, MCP-1 expression was rarely observed in microglia. Following SE, MCP-1 expression was significantly increased in resident IB4 microglia (Figure 2C,D). The fraction of MCP-1 positive cell in total microglia was 70.8% ± 6.2% (Figure 2E). CDDO-Me effectively decreased MCP-1 expression to 0.23 ± 0.04-fold of vehicle level in microglia (*p* < 0.05 vs. vehicle, Student *t*-test, *n* = 7, respectively; Figure 2C,D). Thus, the fraction of MCP-1 positive cell in total microglia was decreased to 23.6 ± 5.6% (*p* < 0.05 vs. vehicle, Student *t*-test, *n* = 7, respectively; Figure 2E). These findings indicate that CDDO-Me-induced microglial inhibition may ameliorate SE-induced monocyte infiltration by reducing MCP-1 synthesis.

### 3.3. CDDO-Me Abolishes Microglial MCP-1 Production Independent of Nrf2 Activation Following SE

**Figure 3:**
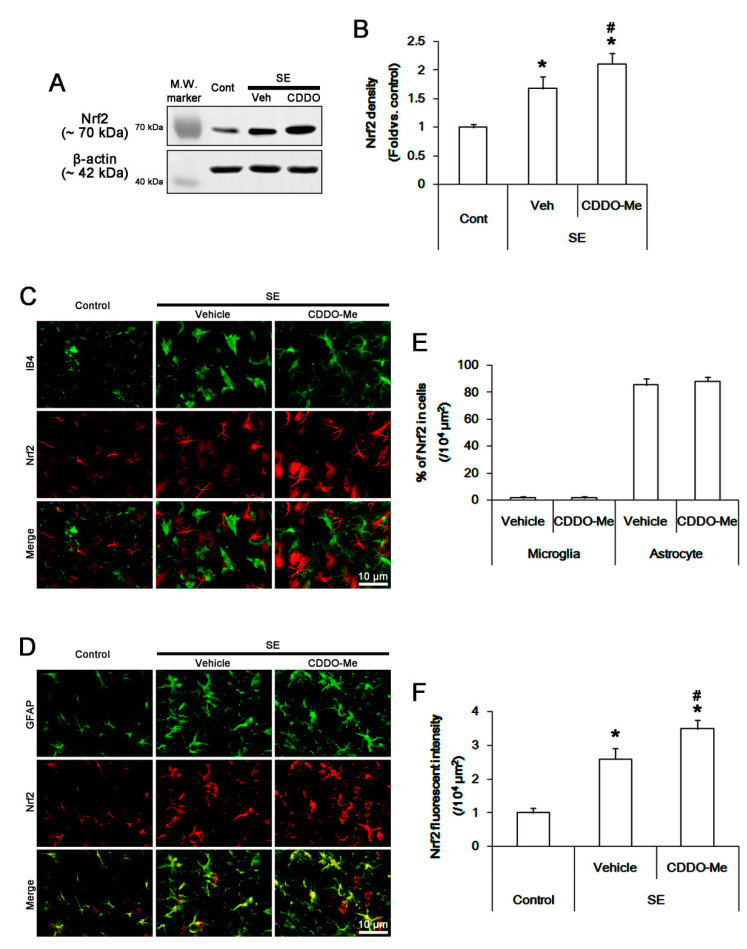
The effect of CDDO-Me on Nrf2 expression following SE. SE increases Nrf2 expression in the FPC, which is more enhanced by CDDO-Me. Nrf2 expression is mainly detected in neurons and astrocytes, but not IB4 microglia, in control animals. SE increases Nrf2 expression in neurons and reactive astrocytes, but not microglia. CDDO-Me more enhances Nrf2 fluorescent intensity. (**A**) Representative Western blot image for Nrf2. (**B**) Quantification of the effect of CDDO-Me on Nrf2 expression based on Western blot data. Error bars indicate SEM (*^,#^ *p* < 0.05 vs. control and vehicle, respectively; *n* = 7, respectively). (C and D) Representative images for Nrf2 expression in IB4 microglia (**C**) and astrocytes (**D**). (E and F) Quantification of the effect of CDDO-Me on the fractions of Nrf2 positive cells in total microglial and astrocytes (**E**) and Nrf2 expression in the FPC (**F**) following SE. Horizontal bars indicate the mean value. Error bars indicate SEM (*^,#^ *p* < 0.05 vs. vehicle; *n* = 7, respectively).

As CDDO-Me reduces MCP-1 production in blood immune cells and monocytes via Nrf2 activation [41], we validated whether CDDO-Me inhibits microglial MCP-1 production through Nrf2-mediated pathway. Western blot data revealed that SE increased Nrf2 protein level to 1.7 ± 0.2-fold of control level in the FPC (*p* < 0.05 vs. control, one-way ANOVA, *n* = 7, respectively; Figure 3A,B). CDDO-Me more enhanced the SE-induced Nrf2 up-regulation to 2.1 ± 0.2-fold of control level (*p* < 0.05 vs. vehicle, one-way ANOVA, *n* = 7, respectively; Figure 3A,B). In control animals, Nrf2 expression was mainly observed in neurons and astrocytes, but not microglia, in the FPC (Figure 3C,D). SE increased Nrf2 fluorescent intensity to 2.6 ± 0.2-fold of the control level without altering the fractions of Nrf2 positive cells in total microglia and astrocytes (*p* < 0.05 vs. control, Student *t*-test, *n* = 7, respectively; Figure 3C–F). CDDO-Me enhanced Nrf2 fluorescent intensity in the FPC more than vehicle following SE (*p* < 0.05 vs. vehicle, one-way ANOVA, *n* = 7, respectively; Figure 3C,D,F). However, CDDO-Me did not influence microglial Nrf2 expression (Figure 3C). These findings indicate that CDDO-ME may attenuate SE-induced microglial activation and monocyte infiltration in Nrf2-independent manners.

### 3.4. CDDO-Me Inhibits P38 MAPK Phosphorylation in Microglia Following SE

**Figure 4:**
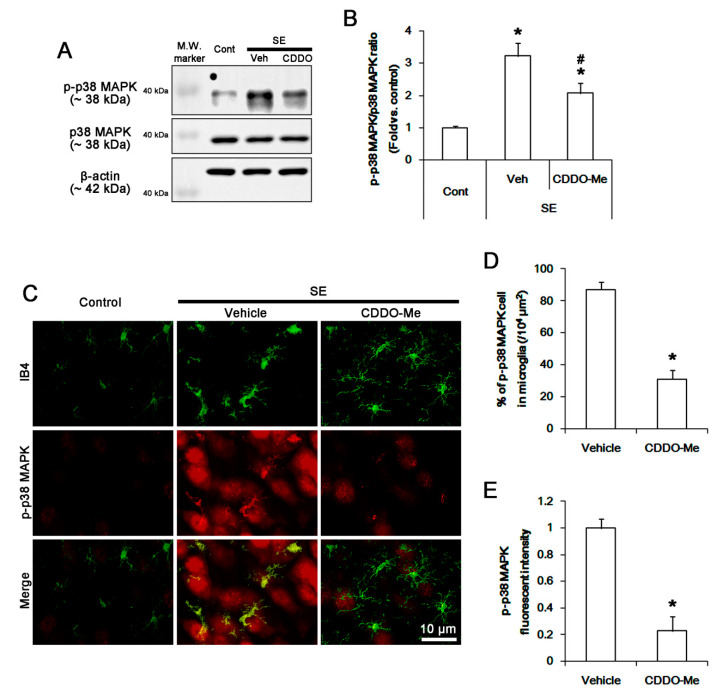
The effect of CDDO-Me on p38 MAPK phosphorylation in microglia following SE. SE increases p38 MAPK phosphorylation in the FPC without altering its expression level, which is ameliorated by CDDO-Me. p38 MAPK phosphorylation is increased in resident IB4 microglia following SE, which is abrogated by CDDO-Me. (**A**) Representative Western blot image for p38 MAPK and p-p38 MAPK. (**B**) Quantification of the effect of CDDO-Me on p38 MAPK phosphorylation based on Western blot data. Error bars indicate SEM (*^,#^ p < 0.05 vs. control and vehicle, respectively; *n* = 7, respectively). (C) Representative images for p38 MAPK phosphorylation in IB4 microglia. (D and E) Quantification of the effect of CDDO-Me on the number of p-p38 MAPK positive cells (**D**) and p-p38 MAPK signals (**E**) following SE. Error bars indicate SEM (* p < 0.05 vs. vehicle; *n* = 7, respectively).

CDDO-Me inhibits p38 MAPK activity [33]. Thus, we investigated whether CDDO-Me influences microglial p38 MAPK activity (phosphorylation) following SE. SE increased p38 MAPK phosphorylation level to 3.2 ± 0.4-fold of control level in the FPC without changing p38 MAPK expression (*p* < 0.05 vs. control, one-way ANOVA, *n* = 7, respectively; Figure 4A,B). CDDO-Me attenuated the SE-induced p38 MAPK phosphorylation to 2.1 ± 0.3-fold of control level (*p* < 0.05 vs. vehicle, one-way ANOVA, *n* = 7, respectively; Figure 4A,B). In control animals, phospho p-p38 MAPK positive cells were rarely detected in the FPC (Figure 4C,D). Following SE, p-p38 MAPK fluorescent intensity was significantly increased in microglia and neurons (Figure 4C,E). Thus, the fraction of p-p38 MAPK positive cells in total microglia was increased to 87.1 ± 4.5% in this region (*p* < 0.05 vs. control, Student *t*-test, *n* = 7, respectively; Figure 4C,D). CDDO-Me decreased the SE-induced p38 MAPK phosphorylation and the fraction of p-p38 MAPK positive cells in total microglia to 0.23% ± 0.1-fold of vehicle level and 31.1 ± 5.6%, respectively (*p* < 0.05 vs. vehicle, Student *t*-test, *n* = 7, respectively; Figure 4C,E). These findings indicate that CDDO-Me may inhibit p38 MAPK activity, which may be relevant to MCP-1 production, following SE. 

### 3.5. CDDO-Me Inhibits NFκB-S276 Phosphorylation in Microglia Following SE

**Figure 5:**
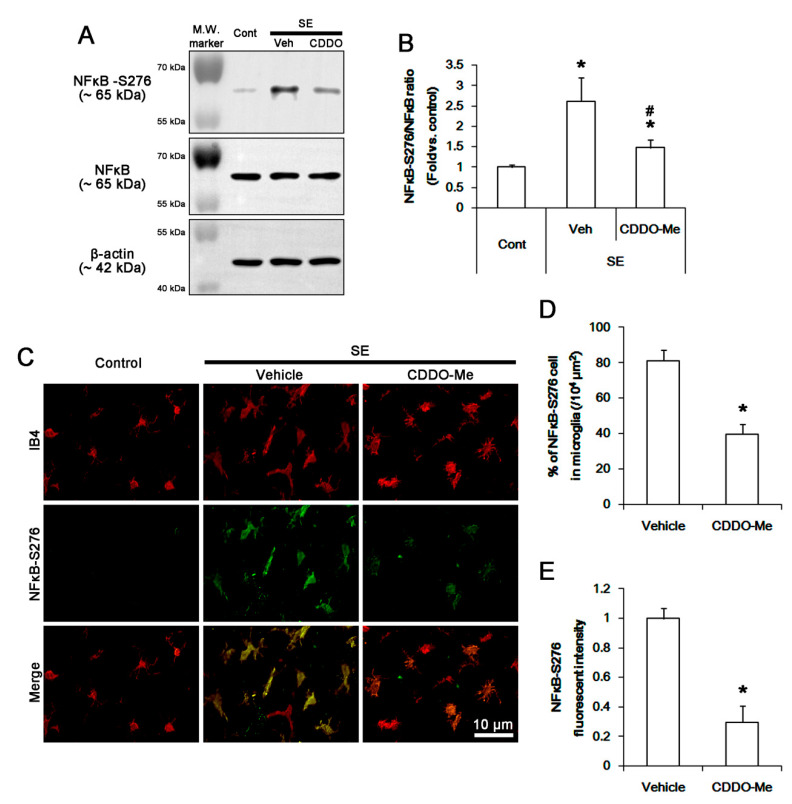
The effect of CDDO-Me on NFκB-S276 phosphorylation in microglia following SE. SE increases NFκB-S276 phosphorylation in the FPC without altering its expression level, which is ameliorated by CDDO-Me. NFκB-S276 phosphorylation is increased in IB4 microglia following SE, while it is rarely observed in control animals. CDDO-Me abolishes NFκB-S276 phosphorylation in IB4 microglia following SE. (**A**) Representative Western blot image for NFκB and NFκB-S276. (**B**) Quantification of the effect of CDDO-Me on NFκB-S276 phosphorylation based on Western blot data. Error bars indicate SEM (*^,#^ *p* < 0.05 vs. control and vehicle, respectively; *n* = 7, respectively). (**C**) Representative images for NFκB-S276 phosphorylation in IB4 microglia. (**D**,**E**) Quantification of the effect of CDDO-Me on the number of NFκB-S276 positive cells (**D**) and NFκB-S276 signals (**E**) following SE. Open circles indicate each value. Horizontal bars indicate the mean value. Error bars indicate SEM (* p < 0.05 vs. vehicle; *n* = 7, respectively).

CDDO-Me inhibits NFκB signaling and the transcription of pro-inflammatory genes including TNF-α [27,36,37]. Since p65 NFκB-S276 phosphorylation is involved in monocyte/microglia-mediated inflammatory responses [47,48], we evaluated the effect of CDDO-Me on its phosphorylation to identify the underlying mechanism of its anti-inflammatory properties. SE increased NFκB-S276 phosphorylation level to 2.6 ± 0.6-fold of control level in the FPC without changing NFκB expression (*p* < 0.05 vs. control, one-way ANOVA, *n* = 7, respectively; Figure 5A,B). CDDO-Me diminished the SE-induced NFκB-S276 phosphorylation to 1.5 ± 0.2-fold of control level (*p* < 0.05 vs. vehicle, one-way ANOVA, *n* = 7, respectively; Figure 5A,B). In control animals, p65 NFκB-S276 signal was rarely observed in microglia (Figure 5C). Following SE, NFκB-S276 fluorescent intensity was elevated in the FPC, thus the fraction of NFκB-S276 positive cells in total microglia was increased to 81.1 ± 6.1% in this region (*p* < 0.05 vs. control, Student *t*-test, *n* = 7, respectively; Figure 5C,E). CDDO-Me effectively ameliorated the up-regulated NFκB-S276 fluorescent intensity and the fraction of p-p38 MAPK positive cells in total microglia to 0.3 ± 0.11-fold of vehicle level and 39.7 ± 5.7%, respectively (*p* < 0.05 vs. vehicle, Student *t*-test, *n* = 7, respectively; Figure 5C,E). 

**Figure 6:**
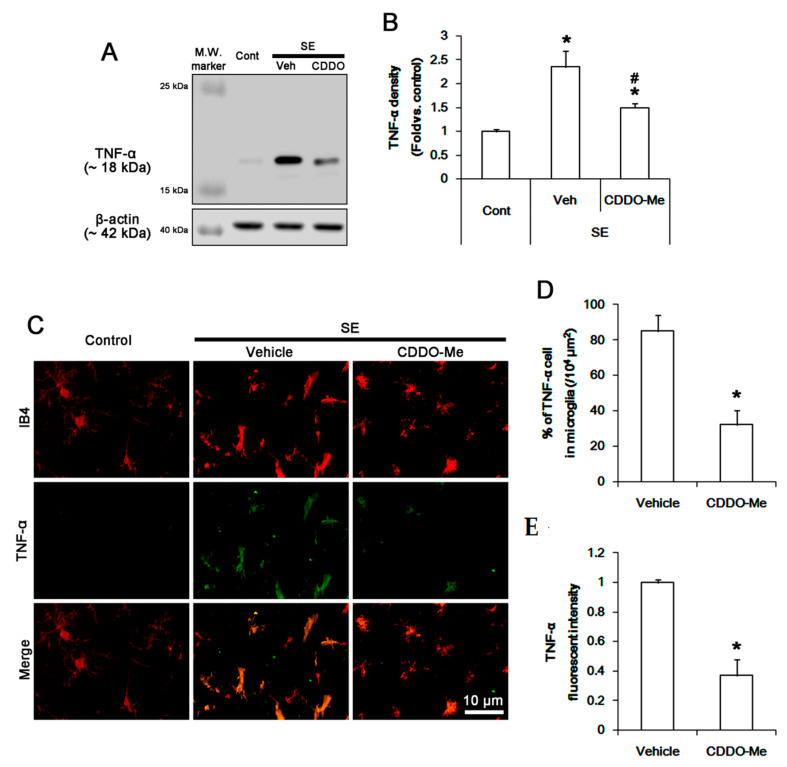
The effect of CDDO-Me on TNF-α in microglia following SE. SE leads to the elevated TNF-α synthesis in the FPC, which is mitigated by CDDO-Me. TNF-α expression is up-regulated in IB4 microglia following SE, while it is rarely observed in control animals. CDDO-Me abolishes TNF-α expression in IB4 microglia following SE. (**A**) Representative Western blot image for TNF-α. (**B**) Quantification of the effect of CDDO-Me on TNF-α induction based on Western blot data. Error bars indicate SEM (*^,#^ p < 0.05 vs. control and vehicle, respectively; *n* = 7, respectively). (**C**) Representative images for TNF-α expression in IB4 microglia. (**D**,**E**) Quantification of the effect of CDDO-Me on the number of TNF-α positive cells (**D**) and TNF-α expression (**E**) following SE. Open circles indicate each value. Horizontal bars indicate the mean value. Error bars indicate SEM (* p < 0.05 vs. vehicle; *n* = 7, respectively).

To confirm the inhibitory role of CDDO-Me in microglial NFκB-S276 phosphorylation, we validated the effect of CDDO-Me on TNF-α synthesis. SE increased TNF-α expression level to 2.4 ± 0.3-fold of control level in the FPC (*p* < 0.05 vs. control, one-way ANOVA, *n* = 7, respectively; Figure 6A,B). CDDO-Me decreased the SE-induced TNF-α induction to 1.5 ± 0.8-fold of control level (*p* < 0.05 vs. vehicle, one-way ANOVA, *n* = 7, respectively; Figure 6A,B). Although TNF-α expression was rarely observed in microglia under physiological condition (Figure 6C), SE increased the fraction of TNF-α positive cells in total microglia to 85% ± 9.1% due to the enhanced TNF-α expression (*p* < 0.05 vs. control, Student *t*-test, *n* = 7, respectively; Figure 6C,E). CDDO-Me abolished the SE-induced up-regulated TNF-α fluorescent intensity and the fraction of TNF-α positive cells in total microglia to 0.37 ± 0.14-fold of vehicle level and 32.1% ± 8%, respectively (*p* < 0.05 vs. vehicle, Student *t*-test, *n* = 7, respectively; Figure 5C,E). Therefore, these findings indicate that CDDO-Me may ameliorate NFκB-mediated microglial activation, which would attenuate monocyte infiltration by inhibiting MCP-1 production following SE.

### 3.6. SN50 Mitigates MCP-1-Mediated Monocyte Infiltration Following SE

**Figure 7:**
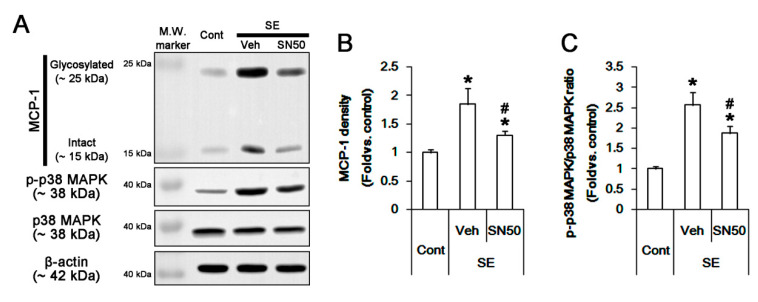
The effect of SN50 on MCP-1 expression and p38 MAPK phosphorylation in the FPC following SE. SN50 decreases MCP1 induction and p38 MAPK phosphorylation without p38 MAPK expression level following SE. (**A**) Representative Western blot image for MCP-1, p-p38 MAPK, and p38 MAPK. (**B**,**C**) Quantification of the effect of SN50 on MCP1 induction (**B**) and p38 MAPK phosphorylation (**C**) based on Western blot data. Error bars indicate SEM (*^,#^ *p* < 0.05 vs. control and vehicle, respectively; *n* = 7, respectively).

**Figure 8:**
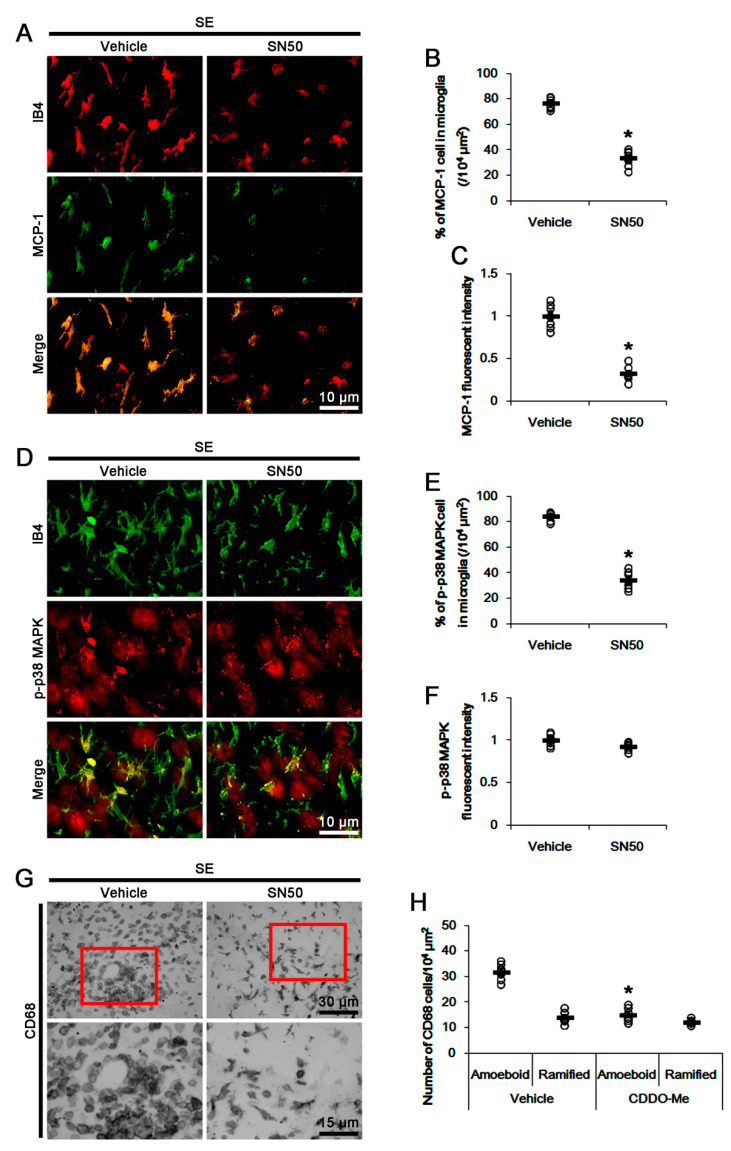
The effect of SN50 on MCP-1 expression, p38 MAPK phosphorylation, and monocyte infiltration following SE. SN50 decreases microglial MCP1 induction, p38 MAPK phosphorylation in microglia, and monocyte infiltration following SE. (**A**) Representative images for microglial MCP-1 expression. (**B**,**C**) Quantification of the effect of SN50 on the number of MCP-1 positive microglia (**B**) and MCP-1 expression (**C**) following SE. (**D**) Representative images for microglial p38 MAPK phosphorylation. (**E**,**F**) Quantification of the effect of SN50 on the number of p-p38 MAPK positive microglia (**E**) and p38 MAPK phosphorylation (**F**) following SE. (**G**) Representative images for CD68 positive cells. (**H**) Quantification of the effect of SN50 on the number of CD68 amoeboid and ramified cells following SE. Open circles indicate each value. Horizontal bars indicate the mean value. Error bars indicate SEM (* *p* < 0.05 vs. control and vehicle, respectively; *n* = 7, respectively).

To confirm the role of NFκB activation in SE-induced MCP-1 induction, we applied SN50 (an NFκB inhibitor) prior to SE. SN50 decreased the SE-induced MCP-1 induction to 0.69-fold of vehicle level (*p* < 0.05 vs. vehicle, one-way ANOVA, *n* = 7, respectively; Figure 7A,B). Immunohistochemical studies revealed that SN50 decreased microglial MCP1 induction following SE (*p* < 0.05 vs. vehicle, Student *t*-test, *n* = 7, respectively; Figure 8A,C). Unexpectedly, SN50 also diminished p38 MAPK phosphorylation level to 0.72-fold of vehicle level following SE (*p* < 0.05 vs. vehicle, one-way ANOVA, *n* = 7, respectively; Figure 7A,C). SN50 inhibited p-p38 MAPK signals in microglia, but not in neurons, and monocyte infiltration following SE (*p* < 0.05 vs. vehicle, Student *t*-test, *n* = 7, respectively; Figure 8D,H). These findings indicate that CDDO-Me may attenuate microglial activation and monocyte infiltration by regulating NFκB- and p38 MAPK-MCP-1 signaling pathways. 

## 4. Discussion

**Figure 9:**
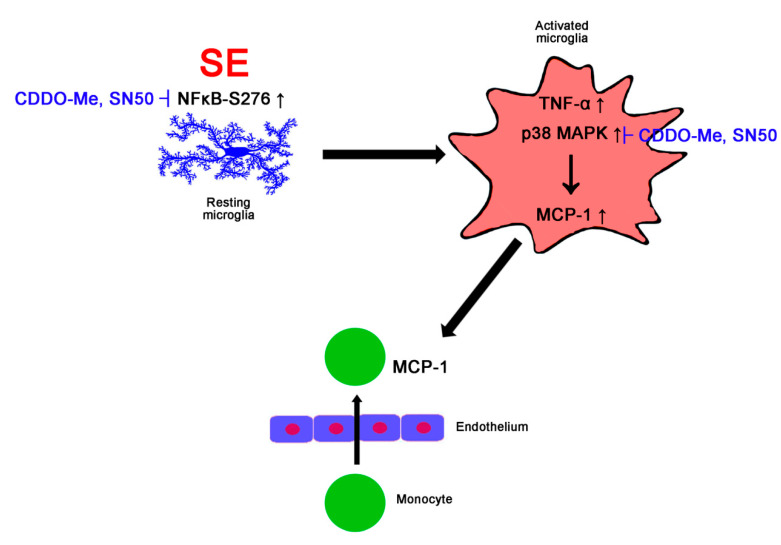
Scheme of the effect of CDDO-Me on monocyte infiltration in FPC following SE. Following SE, the increased NFκB S276 phosphorylation in microglia initiate microglial transformation and up-regulation of TNF-α and MCP-1 expression, which are abolished by CDDO-Me and SN50. In activated microglia, p38 MAPK activation (phosphorylation) also triggers microglial MCP-1 production, which leads to monocyte infiltration. Both CDDO-Me and SN50 inhibits p38 MAPK-mediated MCP-1 expression, which abrogates monocyte infiltration.

In the present study, we found that CDDO-Me ameliorated SE-induced microglial activation and monocyte infiltration in the FPC, accompanied by inhibiting MCP-1 expression and phosphorylation of NFκB-S276 and p38 MAPK, independent Nrf2 activity. Thus, these findings suggest that CDDO-Me may attenuate SE-induced monocyte infiltration and microglial activation by inhibiting NFκB- and p38 MAPK-MCP-1 signaling pathways (Figure 9). 

Microglia are the innate immune effector cells in the central nervous system. Following harmful stresses, microglia change their shape to amoeboid and acquire phagocytic capacity [2]. In addition, activated microglia secrete pro-inflammatory mediators such as TNF-α and MCP-1, which result in monocyte infiltration into the damaged tissue with/without the altered BBB integrity [49]. Together with resident microglia, CD68-positive infiltrating monocytes have the phagocytic ability and aggravate brain lesions by generating ROS, proteolytic enzymes, and pro-inflammatory cytokines [8,11,13,25,50]. Thus, the modulations of microglial activation and monocyte infiltration may ameliorate secondary damage induced by neuroinflammatory responses. In the present study, CDDO-Me inhibited microglia-mediated inflammatory responses to SE insults. Indeed, CDDO-Me prevents high fat diet-induced impairments in recognition memory by reducing inflammation in the PFC [51] and improves neurological functions following focal cerebral ischemia [28,32]. Thus, our findings provide the preclinical evidence concerning the usefulness of CDDO-Me in the treatment/prevention of inflammatory reactions in various neurological diseases. 

The present data reveal that CDDO-ME effectively alleviated SE-induced monocyte infiltration by inhibiting MCP-1 induction. MCP-1 is the first discovered chemokine and functions by recruiting monocytes and T cells to sites of inflammation. CCR2 (the main receptor of MCP-1) is primarily expressed on CD68-positive monocytes [8,25,52,53]. Following SE, resident microglia are the principal cellular sources of MCP-1 [8,10,25]. Recently, we have reported that roscovitine (a cyclin-dependent kinase 5 inhibitor) abrogates SE-induced monocyte infiltration by inhibiting microglial MCP-1 induction without affecting microglial transformation [25]. However, roscovitine did not affect p65 NFκB-S276 phosphorylation and microglial transformation following SE [25]. In the present study, CDDO-Me mitigated monocyte infiltration accompanied by microglial MCP-1/TNF-α inductions. Unlike roscovitine, CDDO-Me inhibited microglial transformation into the elongated/enlarged soma with less ramified processes covering thorny spine following SE. Furthermore, CDDO-Me attenuated p65-Ser276 NFκB phosphorylation induced by SE. As the NFκB inhibition by SN50 abolished microglial transformation in the present study, p65-Ser276 NFκB phosphorylation may likely play an important role in microglial transformation during their activations. 

p65-Ser276 NFκB phosphorylation is required for the microglial MCP-1/TNF-α expressions in microglia/macrophages [31,47,54,55]. The present data demonstrate that both CDDO-ME and SN50 ameliorated MCP-1 induction and monocyte infiltration. As CDDO-Me directly inhibits NFκB signaling [36,37], our findings suggest that CDDO-Me may abrogate NFκB-mediated microglial activation and MCP-1 induction, which would attenuate monocyte infiltration induced by SE. On the other hand, the p38 MAPK signaling pathway is also involved in monocyte infiltration by modulating pro-inflammatory cytokine/chemokine productions including MCP-1 [25,56,57]. Indeed, SB202190, a p38 MAPK inhibitor, ameliorated monocyte infiltration without affecting microglial transformation following SE [25]. In the present study, CDDO-Me reduced phosphorylation of NFκB and p38 MAPK in microglia following SE. As the p38 MAPK pathway is required for NFκB activation [58,59], it is plausible that p38 MAPK-NFκB-MCP-1 would be the main signaling pathway of anti-inflammatory effects of CDDO-Me. In our previous study, however, roscovitine attenuates microglial activation and monocyte infiltration by inhibiting p38 MAPK, independent of NFκB-S276 phosphorylation [25]. Furthermore, inhibition of NFκB-S276 phosphorylation suppresses LPS-induced MCP-1 expression independent of p38 MAPK activity in macrophage [47]. Thus, it is unlikely that CDDO-Me would inhibit microglial activation and monocyte infiltration via the p38 MAPK-NFκB-MCP-1 pathway. Unexpectedly, the present study shows that SN50 attenuated SE-induced MCP-1 induction and p38 MAPK phosphorylation. NFκB indirectly decreases p38 MAPK activity, as NFκB activation up-regulates MKP-1 that dephosphorylates p38 MAPK [60]. In addition, p38 MAPK inhibition does not affect NFκB activation in activated microglia [61]. Thus, it is presumable that SN50-induced NFκB inhibition would reduce p38 MAPK activity via the indirect pathway. However, SN50 also inhibits MAPK kinase (MKK) 3/6 that activates p38 MAPK [61]. Considering these pharmacological properties of SN50, it is a reasonable interpretation that CDDO-Me and SN50 may attenuate SE-induced microglial activation and monocyte infiltration by inhibiting both NFκB and p38 MAPK activity. 

As the modulation of the Nrf2 pathway attenuates microglial activation in vitro [62,63], we explored whether CDDO-Me inhibits SE-induced microglial activation by increasing Nrf2 activity. In the present study, however, CDDO-Me did not affect Nrf2 expression in microglia following SE but increased it in neurons and astrocytes. These findings are consistent with previous studies demonstrating the up-regulation of astroglial Nrf2 expression induced by CDDO-Me [28,31,32]. However, the cell-type-specific expression of Nrf2 is still controversial in vivo: Nrf2 expresses in neurons but not glial cells [64]; in neurons, astrocytes, and microglia [65]; in neurons and astrocytes [28,32]; or in astrocytes [66]. These discrepancies would be the consequence of lower Nrf2 expression in various cells as Kelch-like ECH-associated protein 1 (Keap1) binds to Nrf2 and facilitates Nrf2 degradation via the ubiquitin–proteasome system under physiological condition [67]. CDDO-Me dissociates Keap1 from Nrf2 by interacting with the reactive cysteine 151 residue of Keap1 through a Michael addition [68], which abrogates Keap1-mediated Nrf2 ubiquitination and results in Nrf2 activation [69]. In addition, CDDO-Me itself exerts Nrf2 transcription [70,71]. Regardless of cell-type-specific expression of Nrf2, therefore, our findings suggest that CDDO-Me may attenuate microglia-mediated neuroinflammatory responses independent of Nrf2 activity following SE. On the other hand, CDDO-Me reduced p38 MAPK phosphorylation in both microglia and neurons following SE, although SN50 selectively abolished it in microglia, but not neurons. With respect to the Nrf2-mediated p38 MAPK regulation [72], our findings indicate that CDDO-Me may inhibit p38 MAPK phosphorylation in neurons via Nrf2 activation, independent of NFκB activity. To elucidate the underlying mechanisms of these phenomena, further studies are needed.

## 5. Conclusions

In the present study, we validated, for the first time, the anti-inflammatory effects of CDDO-Me against SE-induced microglial activation and monocyte infiltration (Figure 9). CDDO-Me ameliorated NFκB S276 and p38 MAPK phosphorylation in microglia, which inhibited microglial transformation and TNF-α production. Furthermore, CDDO-Me abolished microglial MCP-1 expression, which mitigated monocyte infiltration. Therefore, these findings propose an underlying pharmacological mechanism of CDDO-Me and its availability for neuroinflammation.

## Figures and Tables

**Table 1:** Primary antibodies and lectin used in the present study.

Antigen	Host	Manufacturer (Catalog Number)	Dilution Used
CD68	Mouse	Abcam (ab31630)	1:100 (IH)
IB4		Vector (B-1205)	1:200 (IH)
Iba-1	Rabbit	Biocare Medical (CP 290)	1:500 (IH)
MCP-1	Mouse	Abcam (ab25124)	1:100 (IH) 1:2000 (WB)
NFκB	Rabbit	Abcam (ab16502)	1:1000 (WB)
NFκB-S276	Rabbit	Abcam (ab106129)	1:100 (IH)
Nrf2	Mouse	Abcam (ab89443)	1:100 (IH) 1:1000 (WB)
p38 MAPK	Rabbit	Cell signaling (#9212)	1:1000 (WB)
phospho-p38 MAPK	Rabbit	Abbiotec (# 251256)	1:200 (IH) 1:500 (WB)
TNF-α	Goat	R&D systems (AF-510-NA)	1:1000 (IH)

IH: Immunohistochemistry; WB: Western blot.

## Supplementary Materials

The following are available online at https://www.mdpi.com/2073-4409/9/5/1123/s1, Figure S1: The whole gel images of Western blot in Figure 2A, Figure 3A and Figure 4A. Figure S2, The whole gel images of Western blot in Figure 5A and Figure 6A. Figure S3, The whole gel images of Western blot in Figure 7A. Table S1: Average of weight and consumptions of food and water in each group.
