# Supplementary material for: CDDO-Me Inhibits Microglial Activation and Monocyte Infiltration by Abrogating NFκB- and p38 MAPK-Mediated Signaling Pathways Following Status Epilepticus
Source: Cells. 2020 May 1;9(5):1123. doi: 10.3390/cells9051123 (PMC7290793; doi:10.3390/cells9051123)
Supplement: Supplementary file 1 [file cells-09-01123-s001.pdf]

Supplementary information

CDDO-Me inhibits microglial activation and monocyte infiltration by abrogating NFκB-p38 MAPK-MCP-1 signaling pathway following status epilepticus

Ji-Eun Kim, Hana Park, Ji-Eun Lee and Tae-Cheon Kang*

Department of Anatomy and Neurobiology, Institute of Epilepsy Research, College of Medicine,

Hallym University, Chuncheon, Kangwon-Do 24252, South Korea[1]

***Correspondence:** Dr. Tae-Cheon Kang, Department of Anatomy and Neurobiology, College of Medicine, Hallym University, Okcheon-dong, Chuncheon, Kangwon-Do, 24252, South Korea, tckang@hallym.ac.kr

Supplementary Table 1. Average of weight and consumptions of food and water in each group

	Group	Weight (g)	Food consumption (g/day)	Water consumption (ml/day)
Control	Vehicle	224 ± 14.8	24 ± 9.4	28 ± 7.1
	CDDO-Me	218 ± 21.3	27 ± 10.1	26 ± 6.8
	SN50	231 ± 18.1	22 ± 7.1	31 ± 5.3
1 day-post SE	Vehicle*	211 ± 24.8	12 ± 8.8	11 ± 8.5
	CDDO-Me*	198 ± 22.4	14 ± 9.4	12 ± 5.7
	SN50*	220 ± 27.9	16 ± 8.3	9 ± 4.8
2 day-post SE	Vehicle	220 ± 18.3	28 ± 5.3	25 ± 6.4
	CDDO-Me	219 ± 7.2	26 ± 4.9	27 ± 5.8
	SN50	212 ± 19.9	31 ± 6.2	28 ± 6.7
3 day-post SE	Vehicle	226 ± 11.7	29 ± 7.1	29 ± 3.3
	CDDO-Me	223 ± 19.4	27 ± 8.7	26 ± 7.3
	SN50	229 ± 21.8	28 ± 6.3	23 ± 6.2

Mean ± SD; No statistical significance in each group.

* Animals were given Hartmann's solution (130 mM NaCl, 4 mM KCl, 3 mM CaCl, and 28 mM lactate; 2 ml/100 g, i.p.)

Fig. 2A

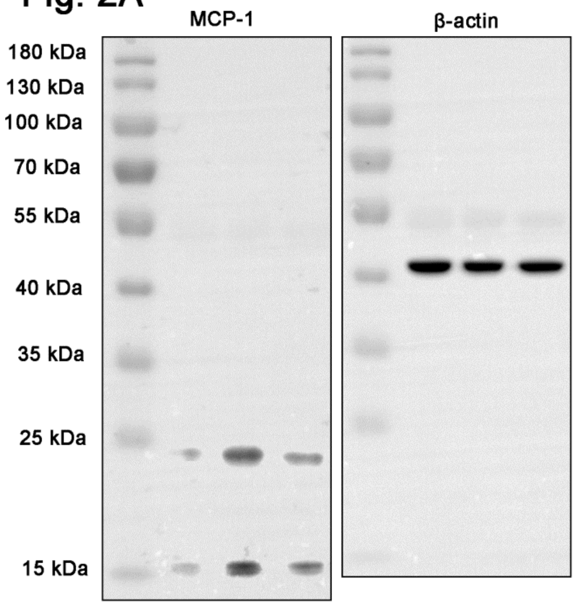

Fig. 3A

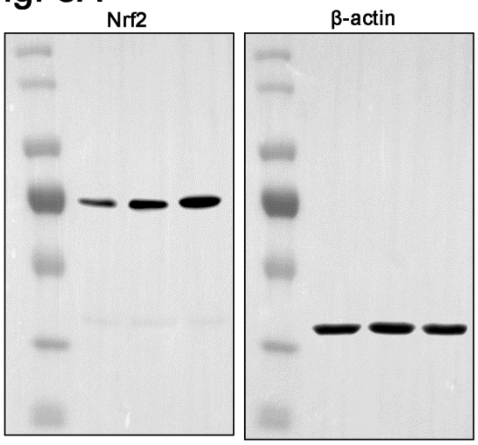

Fig. 4A

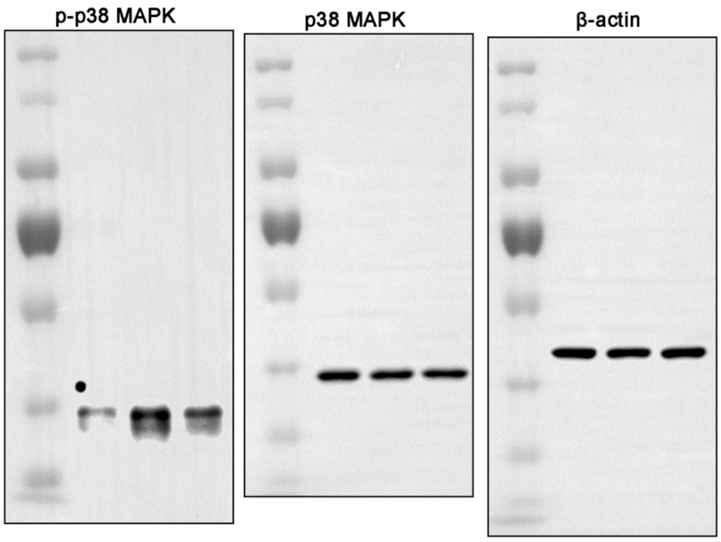

Supplementary Figure 1. The whole gel images of Western blot in Figures 2A, 3A and 4A.

Fig. 5A

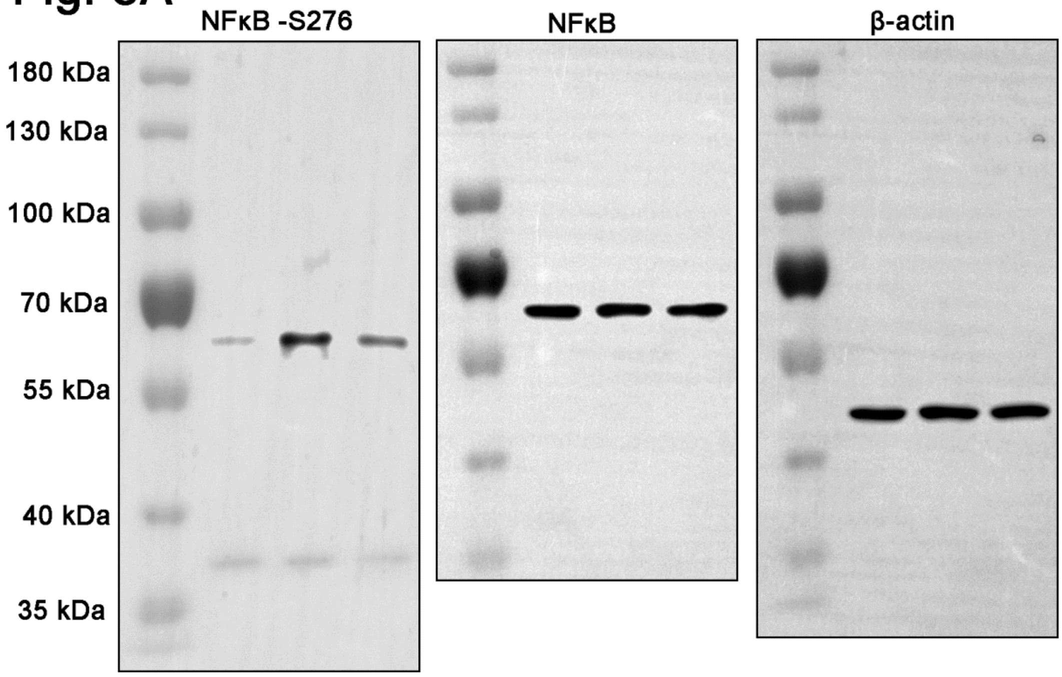

Fig. 6A

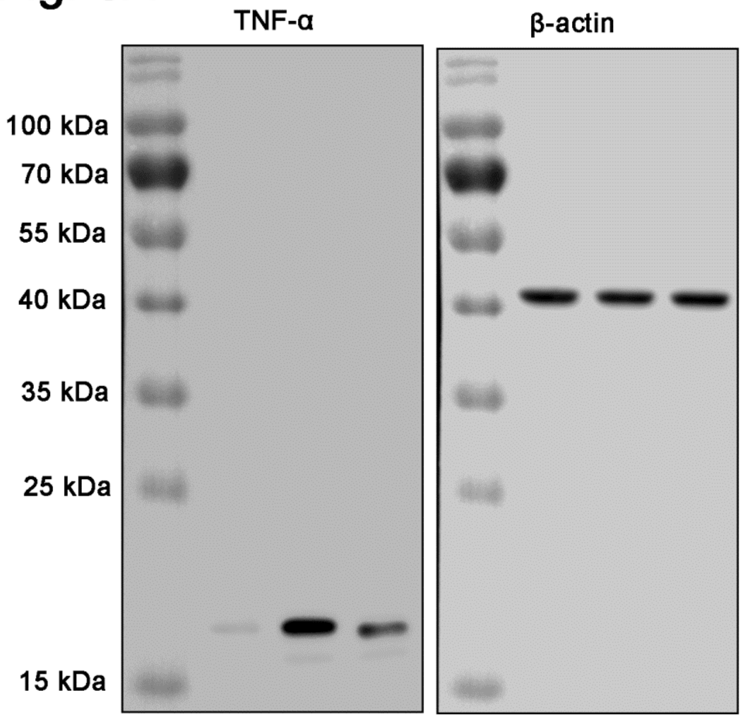

Supplementary Figure 2. The whole gel images of Western blot in Figures 5A and 6A.

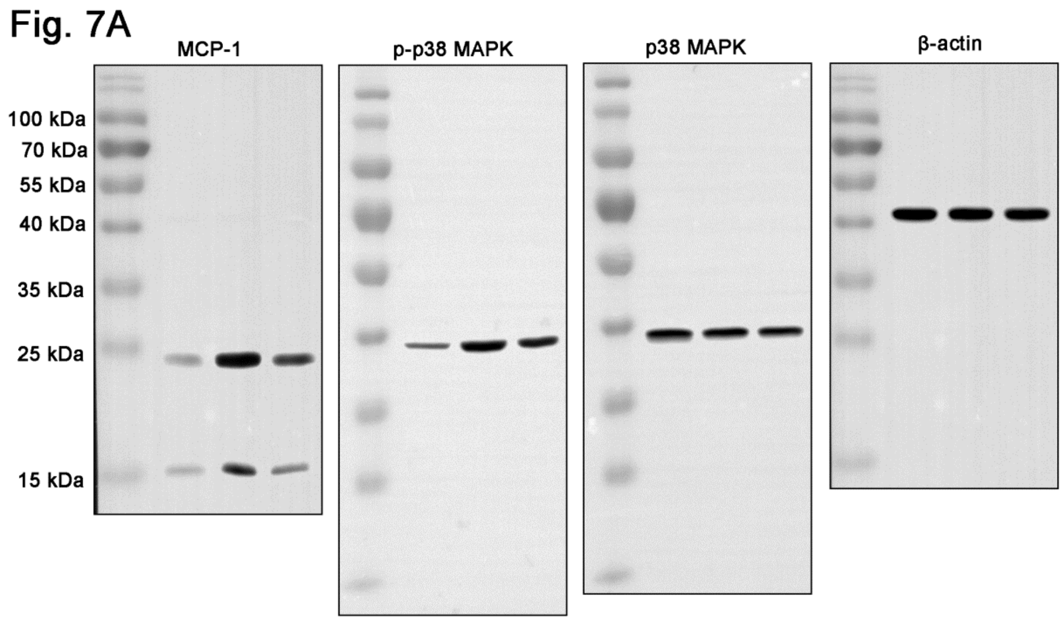

Supplementary Figure 3. The whole gel images of Western blot in Figure 7A.
